# *In vitro* and *in vivo* comparison of the immunotoxicity of single- and multi-layered graphene oxides with or without pluronic F-127

**DOI:** 10.1038/srep38884

**Published:** 2016-12-12

**Authors:** Young Chol Cho, Pyo June Pak, Yong Hoon Joo, Hoi-Seon Lee, Namhyun Chung

**Affiliations:** 1 Department of Biosystems and Biotechnology, College of Life Sciences and Biotechnology, Korea University, Seoul 02841, Korea; 2 College of Agriculture and Life Science, Chonbuk National University, Jeonju 54907, Korea

## Abstract

Graphene oxide (GO) has been a focus of research in the fields of electronics, energy, and biomedicine, including drug delivery. Thus, single- and multi-layered GO (SLGO and MLGO) have been produced and investigated. However, little information on their toxicity and biocompatibility is available. In the present study, we performed a comprehensive study of the size- and dose-dependent toxicity of GOs in the presence or absence of Pluronic F-127 on THP-1 cells by examining their viability, membrane integrity, levels of cytokine and ROS production, phagocytosis, and cytometric apoptosis. Moreover, as an extended study, a toxicity evaluation in the acute and chronic phases was performed in mice via intravenous injection of the materials. GOs exhibited dose- and size-dependent toxicity. Interestingly, SLGO induced ROS production to a lesser extent than MLGO. Cytometric analysis indicated that SLGO induced necrosis and apoptosis to a lesser degree than MLGO. In addition, cell damage and IL-1β production were influenced by phagocytosis. A histological animal study revealed that GOs of various sizes induced acute and chronic damage to the lung and kidney in the presence or absence of Pluronic F-127. These results will facilitate studies of GO prior to its biomedical application.

Graphene exists as single or multi-layered sheets of sp^2^-bonded carbon atoms^1^. The unique physical, chemical, electronic, thermal, and mechanical properties of this carbon-based 2D nanomaterial have attracted interest in biomedicine, such as cancer molecular imaging^2^, enzyme adsorption^3^, biosensors^4^, and drug delivery^5^,^6^. Graphene oxide (GO) is a layered material consisting of hydrophilic oxygenated graphene sheets bearing oxygen functional groups on their basal planes and edges, which facilitates conjugation of proteins^7^, DNAs/RNAs^8^,^9^ or chemical drugs^10^. This property also results in complete exfoliation in water to yield colloidal suspensions of almost entirely individual GO sheets^11^. In addition, compared to graphene, GO is more readily dispersed in water, which makes it more suitable for life science applications than graphene^12^. In addition, multi-layered GO (MLGO), consisting of multiple layers of GO, might have advantages similar to those of multi-walled nanotubes, such as improved resistance to chemicals, large surface area for conjugation, and beneficial mechanical properties^13^, together with the ability of the inner shell to undergo telescopic motion^14^.

Dimensional control on various length scales and individual separation are necessary for interactions between GO and *in vitro* or *in vivo* biological systems^4^. Before application of GO to biological systems, its *in vitro* and *in vivo* immunotoxicity in terms of short- and long-term toxicity of the materials and dose-dependent immunotoxicity should be evaluated^15^,^16^. Currently, GO toxicity has been reported in cytotoxicity and genotoxicity for relatively few layered GO with *in vitro* studies^17^,^18^,^19^,^20^,^21^,^22^,^23^. Thus, we assessed the immunotoxicity of single-layered GO (SLGO) and MLGO against a human monocytic cell line and animal model according to size and dose, exposure time, and the presence of dispersant. We also assessed their renal clearance characteristics.

## Results

### Characterization of GOs for *in vitro* study

The manufacturing company performed an AFM analysis of GOs and provided the product data sheet showing that the topographic heights of SLGO and MLGO were 0.7–1.2 nm and 2.8–3.3 nm, respectively (Supplementary Fig. S1). SLGO and MLGO were of high purity (>99%) and were soluble in water. GOs in solution were sonicated for various time periods to generate a variety of sizes. This is because the size of GO sheets can be decreased by sonication without influencing the chemical reduction process^17^,^18^. The average hydrodynamic diameters (D_h_) in RPMI medium are shown in Supplementary Table S1. Regardless of type of particle and solution, D_h_ values decreased with increasing sonication duration. The water stability of GOs in two solutions was confirmed by determining their zeta-potential values. The zeta-potential values of GOs in RPMI 1640 were –32.4~–46.9 and those in RPMI 1640 containing 1% Pluronic F-127 were –14.0~–23.4. This suggests that the stability of GOs was affected by the physicochemical properties of the solution, which were themselves dependent on the presence of Pluronic F-127.

GO strongly interacts with fetal bovine serum (FBS) proteins^24^. When two sizes (those sonicated for 1 and 15 min) of SLGO and MLGO were incubated with FBS, the supernatant FBS protein level decreased with increasing GO concentration (Supplementary Fig. S2). Interestingly, the FBS protein level in SLGO decreased to a greater degree than that in MLGO. Additionally, smaller GOs (SLGO-15 and MLGO-15) had slightly greater interactions with FBS than did larger GOs (SLGO-1 and MLGO-1).

### GOs-induced cytotoxicity

**Figure 1:**
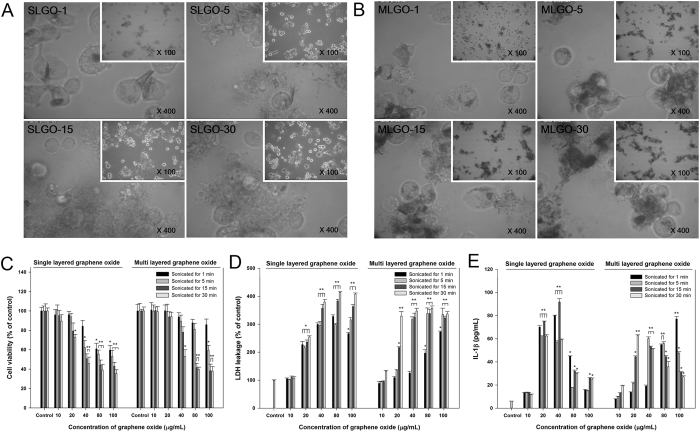
Optical micrographs and cytotoxicity of SLGO- or MLGO-treated PMA-primed THP-1 cells. PMA-primed THP-1 cells were treated with 50 μg/mL of SLGOs or MLGOs of different sizes for 6 h. Cells were visualized by light microscopy to assess their morphology. (**A**) SLGO, (**B**) MLGO. The microscopic image of ‘Control’ was represented at Supplementary Fig. S3. Cytotoxicity induced by SLGOs or MLGOs of different sizes on PMA-primed THP-1 cells. (**C**) WST-1 assay. (**D**) LDH leakage. (**E**) Proinflammatory cytokine (IL-1β) production. Data are the mean ± SE of 3 independent experiments; *p < 0.05, **p < 0.01.

To assess the cytotoxicity of GOs, morphologies of PMA-primed THP-1 cells were observed by optical microscopy after treatment with SLGO and MLGO of different sizes (Fig. 1A,B and Supplementary Fig. S3). PMA is phorbol 12-myristate 13-acetate which conducts the differentiation of THP-1 monocytes into macrophages. Cells treated with larger SLGO-1 and MLGO-1 exhibited little interaction between cells and GOs. In contrast, smaller SLGOs and MLGOs exhibited markedly greater interactions and aggregation. This suggests that aggregation of the cells around GOs induced changes in their morphology.

A WST-1 assay was performed to assess cell proliferation in the presence of GOs of different sizes (Fig. 1C). The degree of cell viability was dependent on GO size and concentration. The decrease in cell viabilities was greater with smaller GOs (SLGO-15, −30 and MLGO-15, -30) than larger GOs (SLGO-1, -5 and MLGO-1, -5). Moreover, SLGOs have higher cytotoxicity than MLGOs. However, the decrease in cell viability was less marked with MLGO-1 and -5 than with SLGO-1 and -5 at >80 μg/mL.

When the cell membrane is damaged, Lactate Dehydrogenase (LDH) molecules are released into the medium^25^. Hence, the LDH level in the culture medium is an indicator of cell membrane damage. With the exception of 10 μg/mL SLGO and MLGO, LDH levels were higher than the control (Fig. 1D). The LDH levels in the SLGO-treated groups were higher than those in the MLGO-treated groups. The LDH level peaked at >40 μg/mL SLGO and MLGO, with the exception of MLGO-1, which resulted in a dose-dependent increase in LDH level. Lysosomal membrane damage or destabilization due to necrotic membrane damage can be examined by acridine orange staining of cells (Supplementary Fig. S4). The degree of lysosomal destabilization was markedly less with SLGO-1 than with SLGO-30. A similar phenomenon was observed with MLGO-1 and MLGO-30. Additionally, the degree of damage was greater with SLGOs than with MLGOs. Thus, smaller GOs resulted in more membrane damage than did larger GOs. This observation coincides with the trends in cell viability and LDH levels.

To evaluate the correlation between treatment with GOs and inflammatory effects, interleukin (IL)-1β production was investigated in the presence of GOs of various sizes and types. All GO treatments stimulated IL-1β production (Fig. 1E). Unlike the trends in the WST-1 and LDH data, the degree of IL-1β production increased rapidly and then decreased at >40 μg/mL. This phenomenon might be due to cell death at >40 μg/mL (Fig. 1C). However, IL-1β production increased in dose-dependent manner following treatment with MLGO-1. This might be correlated with the relatively low cytotoxicity of MLGO-1 (Fig. 1C,D). No specific trend in IL-1β production was observed at the same concentration among GOs of different sizes, with the exception of 10 and 20 μg/mL MLGOs, at which the degree of IL-1β production increased with increasing MLGO size.

### Correlation between reactive oxygen species (ROS) generation and apoptosis

**Figure 2:**
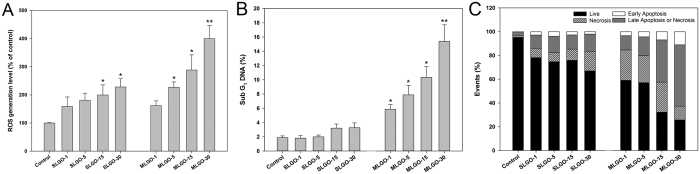
ROS generation and apoptotic cell death induced by SLGOs or MLGOs of different sizes in PMA-primed THP-1 cells. (**A**) ROS generation was measured by loading the cells with DCFH-DA (100 μM) for 30 min, followed by measurement of fluorescence intensity by fluorometer. (**B**) Sub-G1 populations (apoptotic cells) were determined by flow cytometry after PI staining. (**C**) The percentages of apoptotic and necrotic cells were determined from the quadrants of Supplementary Fig. 5. Data are the mean ± SE of 3 independent experiments; *p < 0.05, **p < 0.01.

ROS generation is commonly involved in the toxicological mechanism of nanoparticles and is directly related to apoptosis^26^. Exposure of PMA-primed THP-1 cells to various SLGOs and MLGOs of equal concentrations induced size-dependent oxidative stress, as indicated by the degree of ROS generation (Fig. 2A). ROS generation increased more rapidly with MLGOs than with SLGOs.

The sub-G1 fraction of cell cycle corresponds to cells with fragmented DNA and it can be used as a measure of apoptotic damage^27^,^28^. In cells treated with equal concentrations of SLGOs, the proportion of sub-G_1_ DNA increased only slightly as size decreased (Fig. 2B). However, the proportion of sub-G_1_ DNA upon MLGO treatment was markedly higher than that with SLGOs, and increased in a GO size-dependent manner.

To investigate the mode of cell death upon treatment with GOs, the cells were exposed to equal concentrations of GOs and stained with Annexin V-FITC and PI for flow cytometric analysis. The degree of necrosis and apoptosis increased very slowly with decreasing SLGO size (Fig. 2C and Supplementary Fig. S5). In contrast, the degree of necrosis and apoptosis increased rapidly in a size-dependent manner (Fig. 2A,B).

### Correlation of cell death with phagocytosis

**Figure 3:**
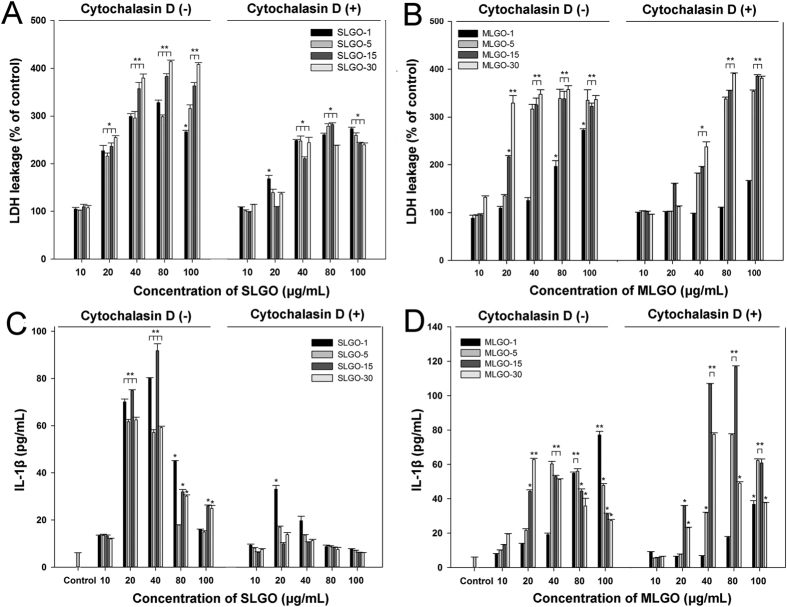
LDH leakage and IL-1β production after treatment with SLGOs or MLGOs with or without a phagocytosis inhibitor. PMA-primed THP-1 cells were treated with SLGOs or MLGOs of different sizes for 6 h in the absence or presence of cytochalasin D (5 μM). (**A**) SLGO, LDH leakage. (**B**) MLGO, LDH leakage. (**C**) SLGO, IL-1β production. (**D**) MLGO, IL-1β production. Data are the mean ± SE of 3 independent experiments; *p < 0.05, **p < 0.01.

Phagocytosis of foreign particles by macrophages causes cell death and initiates innate immune responses, such as IL-1β secretion^29^. To investigate whether the main cause of cell death was phagocytosis or necrotic membrane damage, ELISA and LDH assay were performed. Treatment of cytochalasin D, phagocytosis inhibitor, reduced the degree of LDH leakage in the SLGO-treated groups at all concentrations compared, to the controls (Fig. 3A). These results indicate that SLGO-induced cell death could be caused by membrane damage as well as phagocytosis. Upon treatment with cytochalasin D, the increase in LDH damage slowed with increasing MLGOs concentration (Fig. 3B). However, LDH leakage in the MLGO-treated groups at high doses (>80 μg/mL) were almost identical to the controls. This suggests that phagocytosis does not mediate the cellular toxicity of MLGOs at high concentrations. Cytochalasin D treatment almost completely abolished IL-1β production after SLGOs treatment (Fig. 3C). However, in the presence of MLGOs, IL-1β production was greater with than without cytochalasin D treatment, with the exception of MLGO-1 at 10 and 20 μg/mL. IL-1β production peaked at different concentrations of MLGOs (Fig. 3D). These results suggest that binding and phagocytic uptake of SLGOs cause greater cellular damage and inflammatory responses than those of MLGOs.

### LDH leakage induced by GOs in the presence of Pluronic F-127

**Figure 4:**
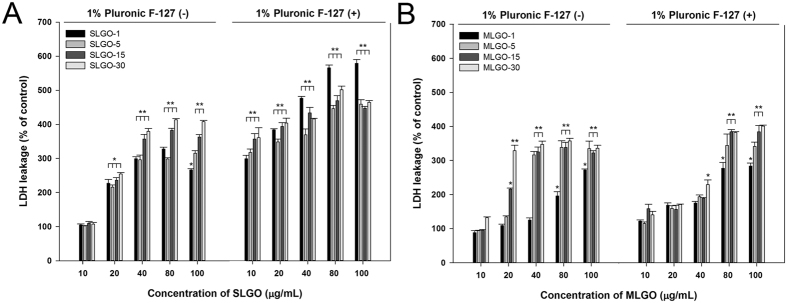
LDH leakage after treatment with SLGOs or MLGOs with or without 1% Pluronic F-127. PMA-primed THP-1 cells were treated with SLGOs and MLGOs of different sizes in RPMI 1640 medium or RPMI 1640 medium containing 1% Pluronic F-127 for 6 h. The control groups incubated in RPMI 1640 or RPMI 1640 containing 1% Pluronic F-127 are not presented as the data are presented as % of the control. (**A**) SLGO. (**B**) MLGO. Data are the mean ± SE of 3 independent experiments; *p < 0.05, **p < 0.01.

Pluronic F-127 (i.e., poloxamer) is water soluble and exhibits low toxicity^30^. The ability of Pluronic F-127 to solubilize hydrophobic solutes has aroused significant interest for applications in drug delivery and controlled release. Pluronic F-127 has been successfully used for dispersion of carbon-based materials, such as carbon nanotubes and graphene, in RPMI medium and showed low toxicity against various cell types^31^,^32^,^33^. Moreover, Pluronic F-127 has been approved by the Food and Drug Administration for clinical use^34^. Pluronic F-127 used at <1% (w/v) was not toxic^30^,^35^,^36^. When cells were exposed to SLGOs, the degree of LDH leakage was greater in the presence than in the absence of Pluronic F-127 (Fig. 4A). Surprisingly, in the presence of Pluronic F-127, the degree of LDH leakage at 10 μg/mL was similar to that at >40 μg/mL in its absence. However, when the cells were exposed to MLGOs in the presence of Pluronic F-127, the degree of LDH leakage increased more slowly than in its absence, particularly for larger-sized MLGOs (Fig. 4B). These results indicate that in the presence of Pluronic F-127 SLGOs induce greater cellular membrane damage than do MLGOs, and that cellular damage depends on the presence of a detergent as well as the size, amount, and type of graphene.

### Characterization of GOs for *in vivo* study

The average D_h_ and zeta-potential values in saline or saline containing Pluronic F-127 are shown in Supplementary Table S2. As has been reported previously, smaller GOs can be produced by longer sonication durations. D_h_ values were only slightly affected by the presence of Pluronic F-127. The average zeta-potential values in the presence of Pluronic F-127 were lower than those in its absence.

### Pulmonary and renal immune responses in terms of monocyte chemoattractant protein-1 (MCP-1) expression levels

MCP-1 is a small cytokine of the C-C chemokine family. It recruits immunocytes such as monocytes, memory T cells, and dendritic cells to sites of tissue injury, infection, and inflammation^37^,^38^. To assess acute damage and innate immune responses, the expression levels of MCP-1 were analyzed.

**Figure 5:**
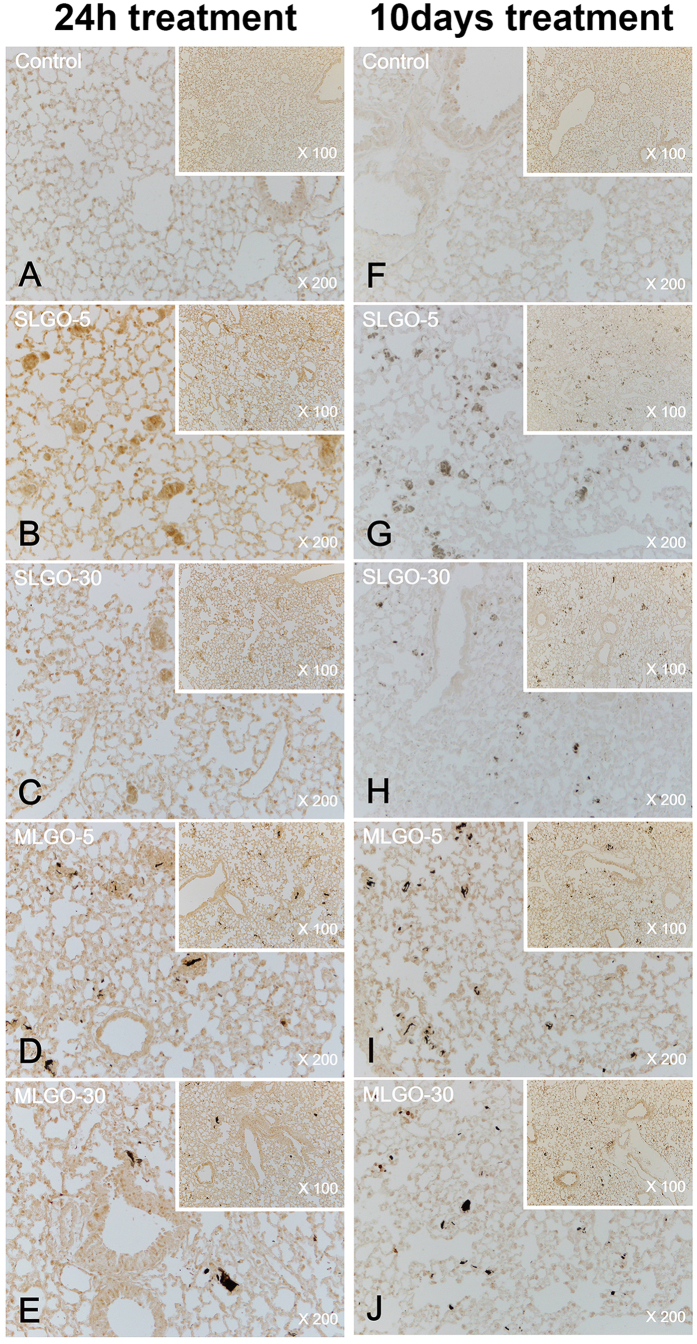
Immunohistochemistry for MCP-1 in the lung during the acute and chronic phases after intravenous injection of SLGOs or MLGOs in saline. At 24 h (acute toxicity evaluation) or 10 days (chronic toxicity evaluation) after injection, MCP-1 expression in the lung was determined. Acute-phase groups: control (**A**), SLGO-5 (**B**), SLGO-30 (**C**), MLGO-5 (**D**), and MLGO-30 (**E**). Chronic-phase groups: control (**F**), SLGO-5 (**G**), SLGO-30 (**H**), MLGO-5 (**I**), and MLGO-30 (**J**).

**Figure 6:**
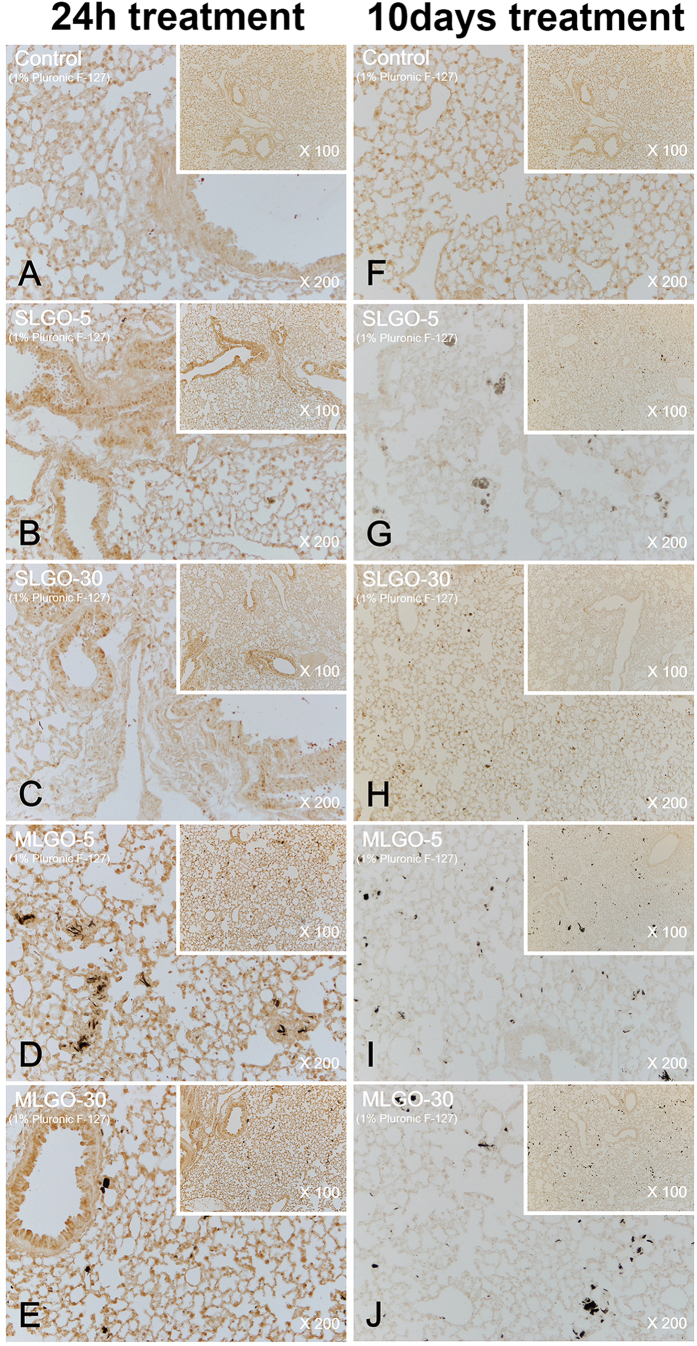
Immunohistochemistry for MCP-1 in lung during the acute and chronic phases after intravenous injection of SLGOs or MLGOs in saline containing 1% Pluronic F-127. At 24 h (acute toxicity evaluation) or 10 days (chronic toxicity evaluation) after injection, MCP-1 expression in the lung was determined. Acute-phase groups: control (**A**), SLGO-5 (**B**), SLGO-30 (**C**), MLGO-5 (**D**), and MLGO-30 (**E**). Chronic-phase groups: control (**F**), SLGO-5 (**G**), SLGO-30 (**H**), MLGO-5 (**I**), and MLGO-30 (**J**).

**Figure 7:**
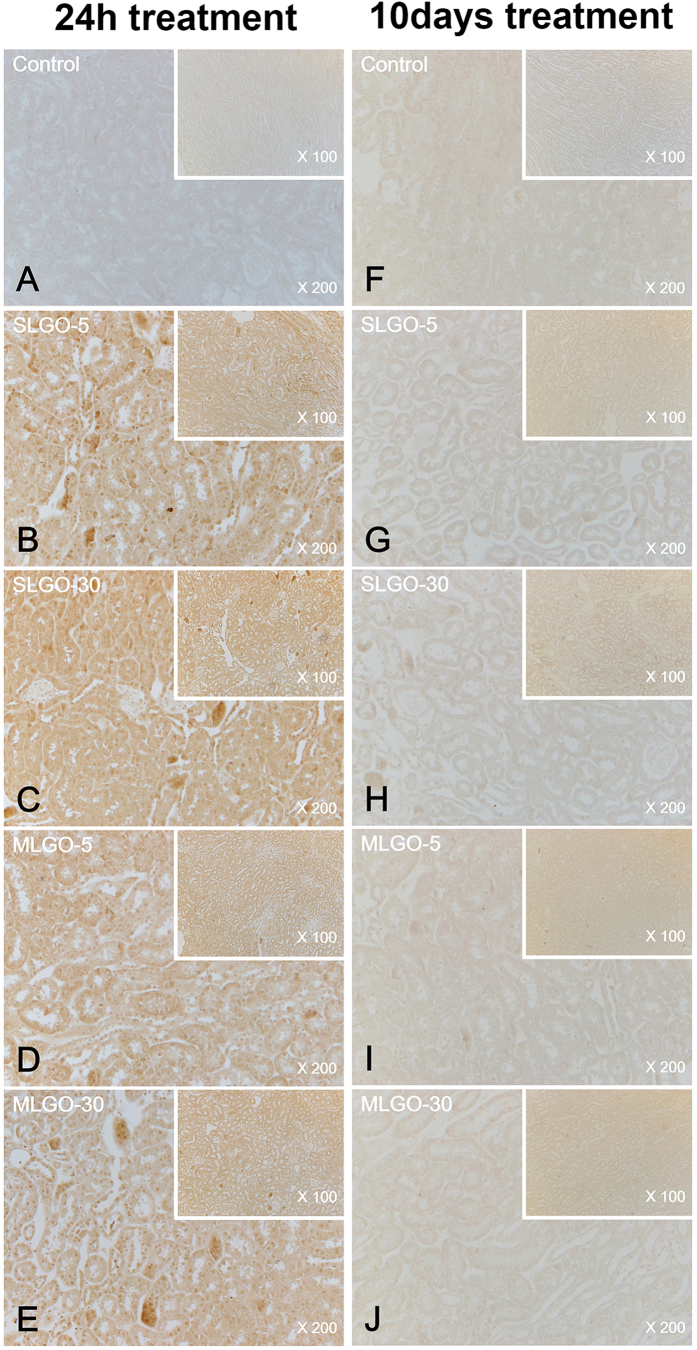
Immunohistochemistry for MCP-1 in the kidney during the acute and chronic phases after intravenous injection of SLGOs or MLGOs in saline. At 24 h (acute toxicity evaluation) or 10 days (chronic toxicity evaluation) after injection, MCP-1 expression in the lung was determined. Acute-phase groups: control (**A**), SLGO-5 (**B**), SLGO-30 (**C**), MLGO-5 (**D**), and MLGO-30 (**E**). Chronic-phase groups: control (**F**), SLGO-5 (**G**), SLGO-30 (**H**), MLGO-5 (**I**), and MLGO-30 (**J**).

**Figure 8:**
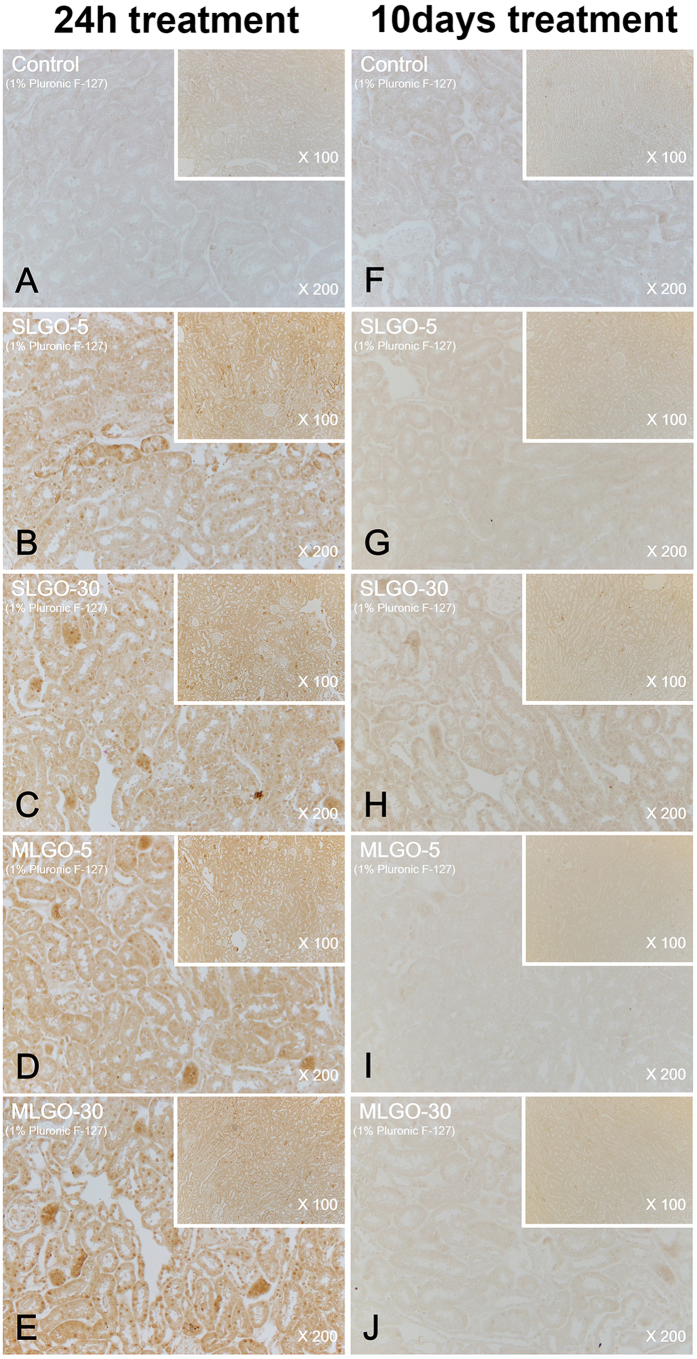
Immunohistochemistry for MCP-1 in the kidney during the acute and chronic phases after intravenous injection of SLGOs or MLGOs in saline containing 1% Pluronic F-127. At 24 h (acute toxicity evaluation) or 10 days (chronic toxicity evaluation) after injection, MCP-1 expression in the lung was determined. Acute-phase groups: control (**A**), SLGO-5 (**B**), SLGO-30 (**C**), MLGO-5 (**D**), and MLGO-30 (**E**). Chronic-phase groups: control (**F**), SLGO-5 (**G**), SLGO-30 (**H**), MLGO-5 (**I**), and MLGO-30 (**J**).

In the acute phase, MCP-1 was expressed in the lung in all groups treated with SLGOs or MLGOs in the presence or absence of Pluronic F-127; the control exhibited no expression (Figs 5 and 6). However, the expression levels did not differ markedly according to GO size and type, or the presence of a dispersant. SLGOs were trapped in lung tissue less frequently than MLGOs. MLGOs were trapped in lung tissue, as indicated by the presence of dark black spots, possibly because of the larger volume of MLGOs than SLGOs (Figs 5 and 6). MCP-1 was also expressed in the kidneys in all GO-treated groups, where GOs were dissolved in saline. As evidenced by dark brown spots, groups treated with SLGO-5, SLGO-30, and MLGO-30 showed higher renal MCP-1 expression levels than the groups treated with MLGO-5 (Fig. 7). This phenomenon might be because MLGO-5 had a larger volume than the other GOs, and so its access to the kidneys would be restricted. MCP-1 expression levels exhibited a similar tendency in the groups treated with GOs in saline containing Pluronic F-127. Unlike the groups treated with MLGO-5 in saline, MCP-1 was expressed throughout the kidneys in all groups, possibly because 1% Pluronic F-127 mediated delivery of the GOs to the kidney following intravenous injection (Fig. 8).

MCP-1 is related to innate immunity and the early phase of inflammation^29^. In the chronic phase, MCP-1 was not expressed in the lungs and kidneys of all treated groups compared to the control groups (Figs 5, 6, 7 and 8). In lungs, although MCP-1 was not expressed, trapped GOs were observed as black spots. These results explain that, in the acute phase, GO treatment induced innate immune responses and inflammation in lung and kidney. Moreover, this phenomenon might be influenced by the size and type of GOs, and the presence of dispersant.

### Renal fibrosis and transforming growth factor (TGF)-β expression levels

To investigate acute and chronic damage due to fibrosis, TGF-β expression was examined. TGF-β is involved in inflammation, autoimmune disorders, cancer, and fibrosis^39^. In the acute phase, TGF-β was not expressed in the lung in all GO-treated groups irrespective of the presence of 1% Pluronic F-127.

However, TGF-β immunoreactivity was found in all GO-treated groups in the chronic phase, likely because TGF-β is related to fibrosis. In the groups treated with SLGOs in saline or saline containing Pluronic F-127, TGF-β was expressed at a low level throughout the kidneys, but there was no marked difference between the groups treated with SLGO-5 or SLGO-30 (Supplementary Figs S6 and S7). In saline, TGF-β immunoreactivity was slightly higher in the MLGO- than the SLGO-treated groups (Supplementary Fig. S6). Indeed, TGF-β expression was highest in the groups treated with MLGOs in saline containing Pluronic F-127. In addition, in the presence of Pluronic F-127, treatment with MLGO-30 resulted in higher TGF-β immunoreactivity than treatment with MLGO-5 (Supplementary Fig. S7). These results suggest that intravenous injection of SLGOs or MLGOs to mice induces fibrosis and inflammation only in the chronic phase, and that addition of Pluronic F-127 increases the degree of fibrosis and inflammation. Moreover, MLGOs induce greater damage than SLGOs and smaller MLGOs cause greater damages and fibrosis than larger MLGOs, irrespective of the presence of Pluronic F-127.

## Discussion

Carbon-based nanomaterials include fullerene, carbon nanotube, carbon nanofiber, carbon nanoparticles, and graphene^40^,^41^,^42^. They have unique thermal, physical, and chemical properties that are potentially useful in biomedicine applications, such as drug and gene delivery^43^,^44^,^45^,^46^. However, the immunotoxicity of GOs has been studied so far^47^,^48^.

Thus, in the present study, the immunotoxicity of GOs was evaluated *in vitro* and *in vivo*. The strong interaction between GOs and proteins was confirmed by BCA assay and sodium dodecyl sulfate polyacrylamide gel electrophoresis (SDS-PAGE)^24^. SLGOs showed higher levels of protein adsorption than MLGOs. Both the type and size of GOs (SLGOs and MLGOs) influenced protein adsorption (Supplementary Fig. S2). We speculated that this difference in protein adsorption might influence immunotoxicity.

The cytotoxicity of SLGOs and MLGOs was investigated *in vitro* using PMA-primed THP-1 macrophages. A WST-1 assay showed that SLGOs and MLGOs exhibit dose-dependent cytotoxicity (Fig. 1C). At >20 μg/mL, smaller GOs exhibited greater cytotoxicity than larger GOs. In addition, MLGOs showed lower cytotoxicity than SLGOs. The LDH assay results showed a similar tendency to those of the WST-1 assay (Fig. 1D). These phenomena were confirmed by acridine orange staining of THP-1 cells exposed to GOs. Smaller GOs (SLGO-30 or MLGO-30) induced higher levels of lysosome membrane destabilization than larger GOs (SLGO-5 or MLGO-5), and the SLGO-treated groups exhibited greater cytotoxicity than the MLGO-treated groups (Supplementary Fig. S4). In contrast, IL-1β production was not dependent on the dose or size of GOs. In the SLGO-treated groups, IL-1β production increased markedly at 20 μg/mL and decreased at >40 μg/mL, suggesting that further increases in GO concentration induced significant necrotic cellular damage (Fig. 1E), as is evident in Fig. 2C. In the MLGO-treated groups, IL-1β production was dose- and size-dependent at low concentrations (10 and 20 μg/mL). The tendency disappeared with further increases in MLGO concentration, possibly due to necrotic cell damage after membrane destabilization.

Nanoparticle toxicity is frequently evaluated by ROS generation and apoptosis induction^26^. GOs induced ROS generation in a size-dependent manner, and MLGOs induced higher levels of ROS generation than SLGOs (Fig. 2A). Moreover, the proportion of sub-G_1_ population in PMA-primed THP-1 cells was increased to a greater extent by MLGOs than SLGOs (Fig. 2B). This suggests that the degree of apoptosis is size-dependent and, more importantly, that apoptosis is caused by ROS generation, the extent of which is affected by the type of GOs. These phenomena were confirmed by flow cytometry, which indicated that the mode of cell death was affected by the type of GO applied (Fig. 2C). These results are in contrast with those in Fig. 1C–E; i.e., the degree of cytotoxicity was higher with SLGOs than with MLGOs. These results suggest that various cytological methods should be used for determination of the cytotoxicity of GOs.

Cell death, as measured by membrane damage, was caused, at least in part, by phagocytosis (Fig. 3A–B). The phagocytosis and IL-1β production results supported this as immune responses, such as IL-1β production by macrophages, can be initiated by phagocytosis of foreign particles (Fig. 3C,D)^27^. Pluronic F-127 has previously been used as a steric stabilizer for nonionic aqueous dispersions of graphene or GO^49^,^50^. In this study, GO cytotoxicity was evaluated in the presence of 1% Pluronic F-127. Surprisingly, addition of this dispersant significantly increased SLGO cytotoxicity in terms of LDH leakage, even at the lowest concentration tested (10 μg/mL) (Fig. 4A). However, at MLGO concentrations <80 μg/mL, the addition of Pluronic F-127 suppressed their cytotoxicity (Fig. 4B). This result is not in agreement with previous reports that graphene or carbon nanotubes hybridized with Pluronic F-127 exhibit no obvious cytotoxicity to MCF-7 or HEK cells^27^,^51^. This suggests use of a dispersant such as Pluronic F-127 should be examined before its application to GO. This is because the pharmacokinetics, short- and long-term fates, and potential toxicity of nanomaterials should be thoroughly examined before use in the clinic^15^,^16^,^52^.

We investigated the acute and chronic *in vivo* toxicity of GOs in lung and kidney after intravenous injection. Irrespective of the presence of Pluronic F-127, when injected intravenously, SLGO and MLGO induced high levels of inflammation in the lungs and kidneys in the acute phase, but their size and number of layers affected the level of inflammation, especially in the kidneys (Figs 5, 6, 7 and 8). These results suggest that even large GOs with multiple layers can reach the kidneys via the lungs and other organs, leading to inflammation, as evidenced by the presence of MCP-1. In addition, as evidenced by the presence of TGF-β, SLGO and MLGO caused various degrees of renal fibrosis and inflammation in the chronic phase (Supplementary Figs S6 and S7). Moreover, treatment of SLGO and MLGO with Pluronic F-127 aggravated renal fibrosis, and smaller MLGOs in Pluronic F-127 induced higher levels of fibrosis than did larger MLGOs. Considering these results and previous reports^53^,^54^,^55^, we speculate that MLGOs in Pluronic F-127 induced higher levels of fibrosis than SLGOs in the chronic phase because MLGOs were thicker and larger, and so they remained in the body for a longer period, or because of stabilization of GOs by Pluronic F-127.

In conclusion, our findings suggest that SLGOs and MLGOs can produce severe cytotoxicity in a dose- and size-dependent manner and induce an immune response, as evidenced by phagocytosis and cytokine expression. Furthermore, the degree of cytotoxicity differed among the various sizes and layer numbers of SLGOs and MLGOs, possibly due to differences in dispersion and protein adsorption. The effects of the size and number of layers should be considered for utilization of SLGOs and MLGOs for drug delivery via intravenous injection, as these influence their deleterious effects, such as immune response and fibrosis. On the other hand, because particle stability might be affected by various latent factors, more research is necessary. For example, although Pluronic F-127 contributed to the stability of carbon-based materials such as GO, the size of GO in the presence of Pluronic F-127 was larger than in the absence of Pluronic F-127. We believe that the phenomenon is due to the depletion force, by which size asymmetry leads to aggregation of relatively large molecules^56^. Moreover, use of Pluronic F-127 to GO solutions in biomedicine should be investigated further to enhances its biocompatibility. The clearance mechanisms of GOs should also be determined. We hope that our results facilitate use of GOs for biomedical applications.

## Methods

### Preparation and characterization of GO for *in vitro* study

GOs were purchased from Cheap Tubes Inc. (Vermont, USA). Characterization of GOs was performed by atomic force microscopy (AFM) analysis, and the manufacturing company provided the product data sheet. To produce a stock solution, GOs were prepared in serum-free RPMI 1640 (WelGENE, Seoul, Korea) at 2 mg/mL and sonicated for 1, 5, 15, and 30 min. Prior to treatment, 10, 20, 40, 80 and 100 μg/mL GOs, were freshly prepared in serum-free RPMI 1640 or RPMI 1640 containing 1% (w/v) Pluronic F-127 (Sigma-Aldrich, Missouri, USA). Various GOs were prepared in the same medium as above at 50 μg/mL GOs for zeta-potential and size measurements, which were performed using a 90 Plus Nanoparticle Size & Zeta-Potential Analyzer (Brookhaven Instruments Co., New York, USA). To examine interactions of GOs with FBS (WelGENE) proteins, GOs were mixed with 5 μL of FBS to produce a 10% (v/v) FBS solution. The mixtures were incubated for 2 h at 37 °C and then centrifuged at 17,000 rcf for 10 min. Each supernatant was subjected to SDS-PAGE, and the gel was visualized by Coomassie brilliant blue staining. FBS concentration in supernatants was determined using a BCA™ Protein Assay Kit (Thermo Scientific, Massachusetts, USA).

### Cell culture and treatment with GO

THP-1 cells (human acute monocytic leukemia cell line; KCLB No. 40202) were obtained from the Korea Cell Line Bank (KCLB, Seoul, Korea) and cultured in RPMI 1640 supplemented with 10% (v/v) heat-inactivated FBS, 0.05 mM 2-mercaptoethanol (Sigma-Aldrich), 100 U/mL penicillin, and 100 μg/mL streptomycin (WelGENE). The culture was maintained at 37 °C in a humidified atmosphere of 5% CO_2_. THP-1 cells were seeded at a density of 5 × 10^5^ cells/mL. The cells were differentiated into macrophage-like cells by adding 0.5 μM of PMA (Sigma-Aldrich) for 24 h prior to treatment with GO^57^. PMA-primed THP-1 cells were washed with Dulbecco’s phosphate-buffered saline (DPBS; WelGENE) and then treated with the indicated concentrations of GOs. Prior to immunotoxicity evaluation, GO-treated cells were incubated for 6 h. For study of phagocytosis inhibition, cytochalasin D (an actin-depolymerizing agent, Sigma-Aldrich) was added to cell monolayers for 30 min prior to treatment with GOs.

### *In vitro* study

To evaluate cell morphology, GO-treated THP-1 cells were gently washed and then images captured by light optical microscopy (CK70, Olympus, Tokyo, Japan). Cell viability was evaluated using a PreMix WST-1 cell proliferation assay (TaKaRa Bio, Shiga, Japan) according to the manufacturer’s protocol^26^,^27^. Membrane integrity was assessed using a LDH Cytotoxicity Detection kit (TaKaRa Bio) according to the manufacturer’s protocol^26^,^27^. We additionally confirmed membrane integrity by acridine orange (Sigma-Aldrich) staining. Briefly, cells were washed three times with pre-warmed DPBS and then stained with 20 μg/mL acridine orange in pre-warmed DPBS for 15 min. Thereafter, the cells were washed and filled with DPBS prior to visualization using a fluorescence microscope (Axio Observer D1, Carl-Zeiss, Oberkochen, Germany). Apoptotic cell death, and ROS and IL-1β production were measured as described previously^26^.

### Preparation for *in vivo* study

Stock solutions of GOs were prepared in saline or saline containing 1% Pluronic F-127, and passed through a 0.22 μm pore size membrane (EMD Millipore Co., Darmstadt, Germany) at 2 mg/mL and then sonicated for 5 or 30 min. To measure zeta-potential and sizes, GOs were prepared in saline or saline containing 1% Pluronic F-127 at 50 μg/mL. Ten-week-old male ICR mice were purchased from Samtako Bio Korea Co. (Osan, Korea). The animals were housed in a conventional state under adequate temperature and humidity (60%) control with a 12 h light/12 h dark cycle (light on at 09:00 h), and all animals were checked for the absence of infection for 7 days prior to use in experiments.

### *In vivo* study

Mice were divided into 20 experimental groups. Thus, the two groups for the evaluation of acute and chronic immunotoxicity each comprised 10 experimental groups. The animals were sacrificed after 24 h (acute toxicity) or 10 days (chronic toxicity) from the time of injection with GOs. The control group received 200 μL of saline or saline containing 1% Pluronic F-127. Groups treated with GOs were received a dose of 10 mg/kg in the same solution as the control group. Mice were anesthetized using a mixture of 30 mg zoletil and 10 mg rompun per kg body weight intraperitoneally and then sacrificed. Before collection of organs, the animals were perfused transcardially with PBS. The left lobes of the lungs and the left kidneys were collected and fixed in 4% paraformaldehyde in PBS to obtain immunohistochemistry (IHC) data for MCP-1 and TGF-β. The paraffin sections (4 μm) were hydrated and treated with 0.3% hydrogen peroxide in PBS for 30 min. For antigen retrieval, the sections were placed in 400 mL jars filled with citrate buffer (pH 6.0) and heated in a microwave oven (Optiquick Compact, Moulinex, Germany). After three heating cycles of 5 min each, slides were allowed to cool to room temperature and were washed in PBS. After washing, the sections were incubated successively in 10% normal rabbit serum in PBS for 30 min and in diluted rabbit anti-MCP-1 (1:100 dilution, Abcam, Cambridge, UK) or rabbit anti-TGF-β (1:200 dilution, Cell Signaling Technology, Massachusetts, USA) at 4 °C for 48 h. Thereafter, they were exposed to biotinylated rabbit anti-goat IgG or goat anti-rabbit IgG, streptavidin peroxidase complex (1:20 dilution, Vector Laboratories, California, USA), and then visualized with 3,3-diaminobenzidine tetrahydrochloride (Sigma-Aldrich) in 0.1 M Tris-HCl buffer.

### Statistical analysis

Data are expressed as means ± standard error (SE). The data were analyzed using Student’s t-test or Mann-Whitney U test and performed by using the SPSS 20.0 software (IBM Corp., Armonk, NY, USA). Statistical significance was determined in level of *p < 0.05 or **p < 0.01.

## Additional Information

**How to cite this article**: Cho, Y. C. *et al*. *In vitro* and *in vivo* comparison of the immunotoxicity of single- and multi-layered graphene oxides with or without pluronic F-127. *Sci. Rep.* **6**, 38884; doi: 10.1038/srep38884 (2016).

**Publisher's note:** Springer Nature remains neutral with regard to jurisdictional claims in published maps and institutional affiliations.

## Supplementary Material

Supplementary Information
