# Supplementary material for: In vitro and in vivo comparison of the immunotoxicity of single- and multi-layered graphene oxides with or without pluronic F-127
Source: Sci Rep. 2016 Dec 12;6:38884. doi: 10.1038/srep38884 (PMC5150857; doi:10.1038/srep38884)
Supplement: Supplementary Information [file srep38884-s1.pdf]

***In vitro* and *in vivo* comparison of the immunotoxicity of single- and multi-layered graphene oxides with or without pluronic F-127**

Young Chol Cho[1,3], Pyo June Pak[1,3], Yong Hoon Joo[1], Hoi-Seon Lee[2,]*, Namhyun Chung[1,]*

[1]Department of Biosystems and Biotechnology, College of Life Sciences and Biotechnology, Korea University, Seoul 02841, Korea. [2]College of Agriculture and Life Science, Chonbuk National University, Jeonju 54907, Korea. [3]Y.C.C. and P.J.P. contributed equally to this study. Correspondence and requests for materials should be addressed to N.C. (email: nchung@korea.ac.kr) and H.-S.L. (email: hoiseon@jbnu.ac.kr)

Keywords: cytotoxicity, immunotoxicity, multi-layered graphene oxide, single-layered graphene oxide

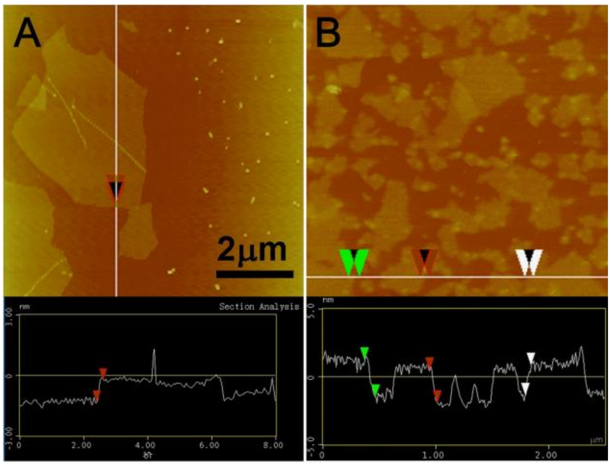

Supplementary Figure S1 AFM topography images of (A) single-layered graphene oxide and (B) multi-layered graphene oxide.

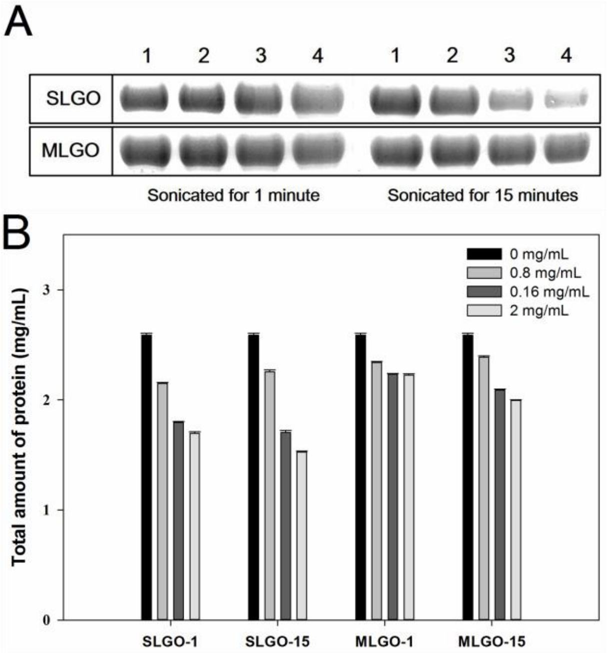

Supplementary Figure S2 Characterization of interaction of SLGOs and MLGOs of different sizes with FBS proteins. (A) SDS-PAGE of FBS proteins in the supernatant after centrifugation. FBS solution (50 μg/mL of 10% (v/v)) was incubated with SLGO or MLGO of different amounts and sizes (lanes 1 to 4: 0, 40, 80, and 100 μg, respectively) for 2 h at 37°C. The bands were located between the 50 and 80 kDs markers. (B) Quantification by BCA assay of total FBS protein in the same supernatant used for (A).

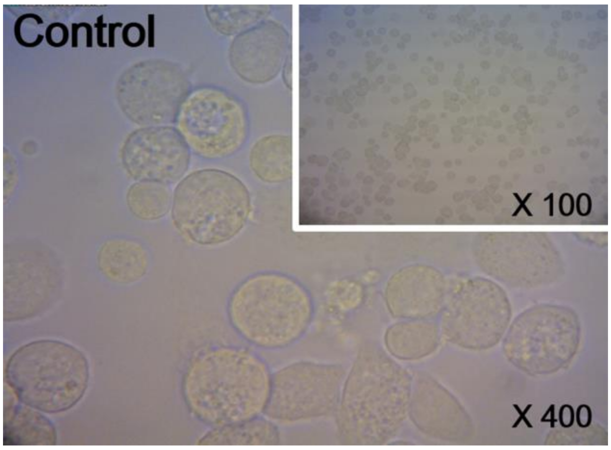

Supplementary Figure S3 Optical micrographs of PMA-primed THP-1 cells.

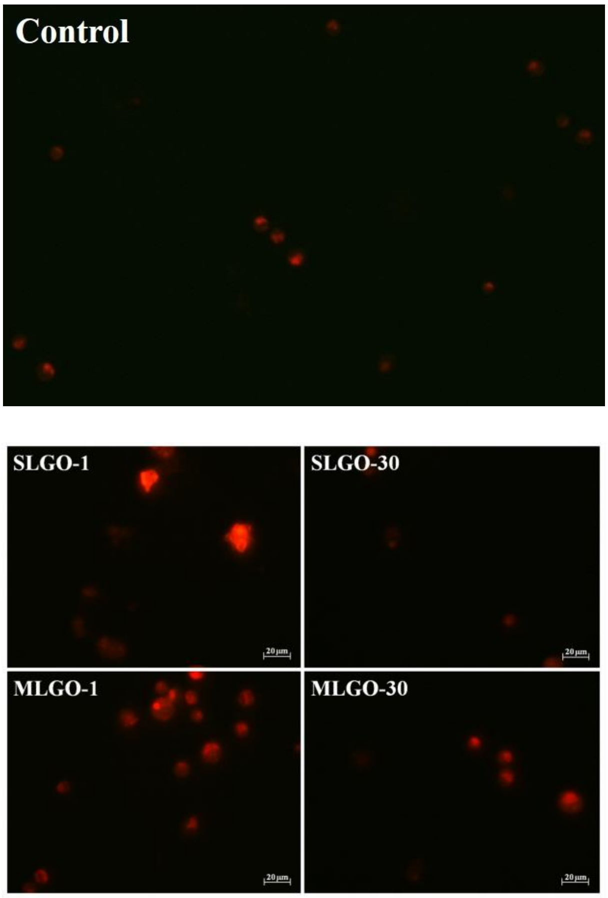

Supplementary Figure S4 Lysosomal destabilization induced by SLGO and MLGO of different sizes. PMA-primed THP-1 cells were treated with graphene oxide particles (50 µg/mL) for 6 h. At the end of exposure, cells were loaded with acridine orange (20 µg/mL) for 15 min at 37°C and imaged using a fluorescence microscope (×400). Acridine orange enters the acidic lysosomal compartments. Release of acridine orange is an indicator of necrotic damage.

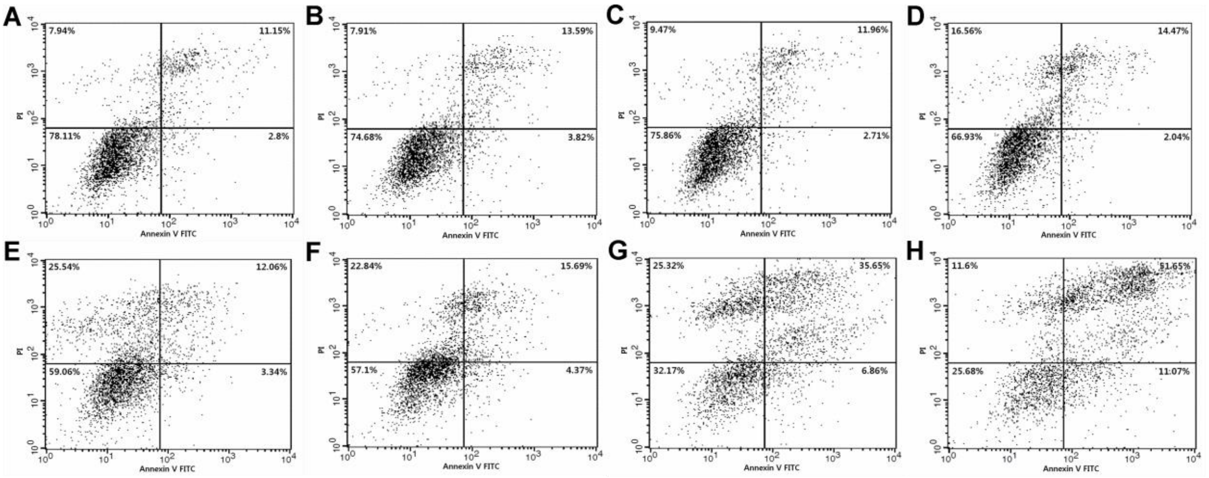

Supplementary Figure S5 Induction of PMA-primed THP-1 cell death by SLGOs and MLGOs of different sizes. Cells were stained with Annexin V and PI and then subjected to flow cytometry. SLGO-1 (A), SLGO-5 (B), SLGO-15 (C), SLGO-30 (D), MLGO-1 (E), MLGO-5 (F), MLGO-15 (G), and MLGO-30 (H).

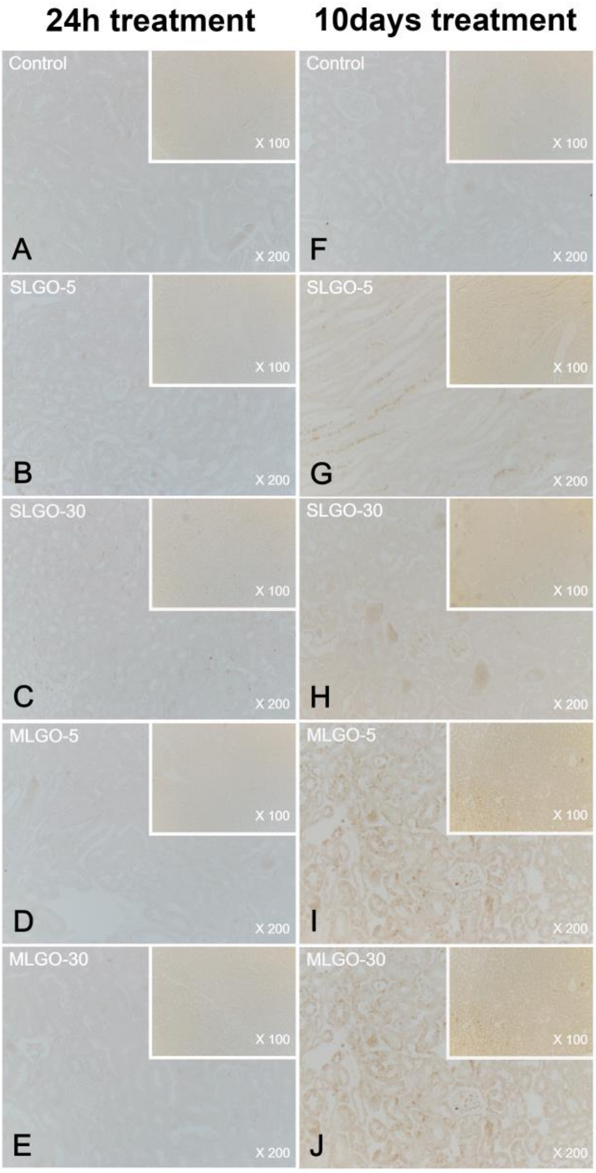

Supplementary Figure S6 Immunohistochemistry of TGF-β in the kidney during the acute and chronic phases after intravenous injection of SLGOs or MLGOs in saline. Acute-phase groups: control (A), SLGO-5 (B), SLGO-30 (C), MLGO-5 (D), and MLGO-30 (E). Chronic-phase groups: control (F), SLGO-5 (G), SLGO-30 (H), MLGO-5 (I), and MLGO-30 (J).

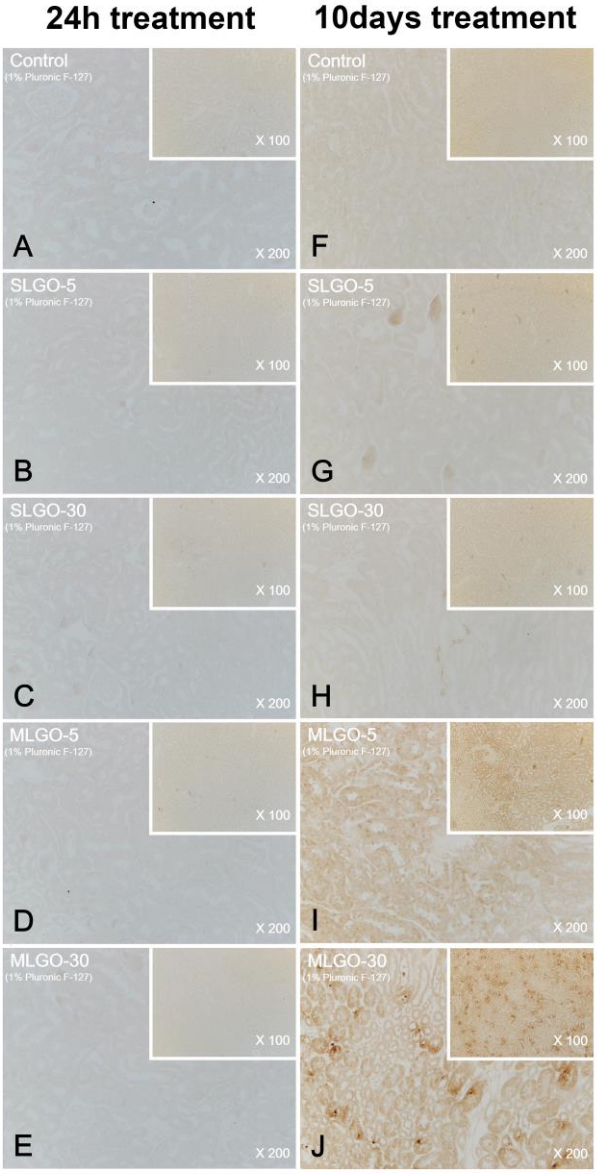

Supplementary Figure S7 Immunohistochemistry of TGF-β in the kidney during the acute and chronic phases after intravenous injection of SLGO or MLGO in saline containing 1% Pluronic F-127. Acute-phase groups: control (A), SLGO-5 (B), SLGO-30 (C), MLGO-5 (D), and MLGO-30 (E). Chronic-phase groups: control (F), SLGO-5 (G), SLGO-30 (H), MLGO-5 (I), and MLGO-30 (J).

Supplementary Table S1 Characteristics of SLGO and MLGO used for the *in vitro* study.

**Samples**	**D_h* in RPMI 1640 (nm)**	**D_h* in RPMI 1640 + 1% Pluronic F-127 (nm)**	**ζ-potential in RPMI 1640 (mV)**	**ζ-potential In RPMI 1640 + 1% Pluronic F-127 (mV)**
**SLGO-1**	878.5±102.6	1010.9±101.8	-39.6±3.5	-20.9±0.1
**SLGO-5**	816.8±40.7	892.0±79.8	-37.3±2.0	-21.9±2.0
**SLGO-15**	699.0±84.0	718.6±92.4	-36.2±5.4	-21.7±2.0
**SLGO-30**	497.1±42.1	657.3±64.2	-44.4±2.0	-23.4±1.6
**MLGO-1**	1259.2±101.9	1148.4±92.2	-32.4±1.8	-19.1±2.6
**MLGO-5**	1156.0±82.5	982.3±46.1	-44.5±3.7	-20.8±0.2
**MLGO-15**	912.2±75.4	805.6±57.7	-46.9±1.9	-22.1±2.0
**MLGO-30**	701.6±109.1	703.0±79.7	-37.0±3.7	-14.0±0.1

* Hydrodynamic diameter (D_h)

Values are means ± standard deviation of triplicate measurements. Samples in RPMI 1640 or RPMI containing 1% Pluronic F-127 were measured at 50 µg/mL.

Supplementary Table S2 Characteristics of SLGO and MLGO for the *in vivo* study.

Samples	D_h* in Saline (nm)	D_h* in Saline + 1% Pluronic F-127 (nm)	ζ-potential in Saline (mV)	ζ-potential In Saline + 1% Pluronic F-127 (mV)
**SLGO-5**	938.6±86.5	996.0±58.8	-34.8±3.0	-31.3±3.5
**SLGO-30**	605.3±65.1	656.1±80.9	-37.3±1.1	-30.5±0.2
**MLGO-5**	1013.3±80.4	1142.6±64.7	-58.8±3.0	-41.5±3.3
**MLGO-30**	651.1±104.5	745.7±16.6	-66.3±3.9	-45.9±1.7

* Hydrodynamic diameter (D_h)

Values are means ± standard deviation of triplicate measurements. Samples in saline or saline containing 1% Pluronic F-127 were measured at 50 μg/mL.
